# CARDIORESPIRATORY FITNESS AND MITOCHONDRIAL ENERGETICS IN THE OLDEST OLD: THE STUDY OF MUSCLE, MOBILITY AND AGING

**DOI:** 10.1093/geroni/igad104.2081

**Published:** 2023-12-21

**Authors:** Giovanna Distefano, John Noone, Sofhia Ramos, Philip Kramer, Peggy Cawthon, Anne Newman, David Marcinek, Bret Goodpaster

**Affiliations:** AdventHealth, Orlando, Florida, United States; AdventHealth, Orlando, Florida, United States; AdventHealth, Orlando, Florida, United States; Wake Forest University School of Medicine, Winston-Salem, North Carolina, United States; California Pacific Medical Center, Research Institute, San Francisco, California, United States; University, Pittburgh, Pennsylvania, United States; University of Washington, Seattle, Washington, United States; AdventHealth, Orlando, Florida, United States

## Abstract

Cardiorespiratory fitness (CRF) and mitochondrial energetics are known to decrease with aging, albeit with a great degree of heterogeneity. Studies reporting these declines typically include individuals younger than 85 years of age. We investigated CRF and mitochondrial energetics in a cohort of old (O: 70-84yrs.; n=698) and oldest old (OO: 85+yrs.; n=70) adults enrolled in SOMMA. Cardiorespiratory fitness (VO2peak) was measured by modified Balke treadmill protocol, and mitochondrial energetics was assessed by 31P Magnetic Resonance Spectroscopy (ATPmax) and high-resolution respirometry of myofibers (oxidative phosphorylation, OXPHOS). Bivariate associations and group differences were assessed using unadjusted and adjusted regression models with clinical site/technician, gender, race, body mass index and physical activity (steps per day) as covariates. Old and oldest old groups showed an association between VO2peak and OXPHOS (R2=0.282, p<0.001; R2=0279, p=0.0083; respectively, adjusted for site/technician), but only the old group showed associations between VO2peak and ATPmax (R2=0.201, p<0.001; R2=0.017, p=0.609; respectively, adjusted for site/technician). Group comparison adjusted for clinical site, gender, and race, showed lower VO2peak (p < 0.001), OXPHOS (p=0.026), and ATPmax (p=0.0014) in the OO compared to O group. However, no group differences in OXPHOS (p=0.08) and ATPmax (p=0.09) are observed when adjusting for body composition and physical activity, factors known to affect mitochondria. In conclusion, CRF associates with different measures of mitochondrial energetics in older adults, but only with mitochondrial respiration in oldest old. Additionally, we suggest that decreases in mitochondrial energetics observed in the oldest old are related to age, changes in body composition and physical activity.

